# Association between P2RY12 Gene Polymorphisms and IVIG Resistance in Kawasaki Patients

**DOI:** 10.1155/2020/3568608

**Published:** 2020-03-10

**Authors:** Zhouping Wang, Yufen Xu, Huazhong Zhou, Yanfei Wang, Wei Li, Zhaoliang Lu, Zhiyong Jiang, Xueping Gu, Hao Zheng, Lanlan Zeng, Ping Huang, Li Zhang, Xiaoqiong Gu

**Affiliations:** ^1^Department of Cardiology, Guangzhou Women and Children's Medical Center, Guangzhou Medical University, Guangzhou, Guangdong, China; ^2^Department of Clinical Lab, Guangzhou Institute of Pediatrics, Guangzhou Women and Children's Medical Center, Guangzhou Medical University, Guangzhou, Guangdong, China; ^3^Department of Blood Transfusion and Clinical Lab, Guangzhou Institute of Pediatrics, Guangzhou Women and Children's Medical Center, Guangzhou Medical University, Guangzhou 510623, Guangdong, China

## Abstract

Children with Kawasaki disease (KD) resistant to intravenous immunoglobulin (IVIG) have a higher incidence of coronary artery lesions (CAL). Despite the association between Purinergic receptor P2Y12 (P2RY12) polymorphism, KD genetic susceptibility, and CAL complications being proved, few studies have assessed the relationship between P2RY12 polymorphisms and IVIG resistance in patients with KD. We recruited 148 KD patients with IVIG resistance and 611 with IVIG sensitivity and selected five P2RY12 polymorphisms: rs9859538, rs1491974, rs7637803, rs6809699, and rs2046934. A significant difference in the genotype distributions between patients was only observed for the rs6809699 A > C polymorphism (AC vs. AA: adjusted odds ratio (OR) = 0.48, 95% confidence interval (CI) = 0.27–0.84, *P*=0.011; AC/CC vs. AA: adjusted OR = 0.47, 95% CI = 0.27–0.83, *P*=0.0084). After adjusting for age and gender, the carriers of the rs6809699 C allele had OR of 0.44 to 0.49 for IVIG sensitivity (AC vs. AA: adjusted OR = 0.48, 95% confidence interval (CI) = 0.27–0.84, *P*=0.011; AC/CC vs. AA: adjusted OR = 0.47, 95% CI = 0.27–0.83, *P*=0.0084) compared to the carriers of a rs6809699 AA genotype, suggesting the protective effect of this SNP against IVIG resistance. Moreover, individuals with all five protective polymorphisms experienced a significantly decreased IVIG resistance compared to that of individuals with up to three protective polymorphisms (adjusted OR = 0.27, 95% CI = 0.13–0.57, *P*=0.0006). Our results suggest that the P2RY12 rs6809699 polymorphism could be used as a biomarker to predict IVIG resistance in KD patients.

## 1. Introduction

Kawasaki disease (KD) is an acute, self-limited vasculitis of unknown etiology that occurs predominantly in infants and children [1]. KD leads to coronary artery aneurysms in approximately 25% of untreated cases [2]. Studies have shown that even if there is no CAL in the acute phase of KD, there is still a risk of developing arterial endothelial dysfunction, increased vascular stiffness, and decreased myocardial reserve capacity in adolescent and adult patients [3, 4]. At present, KD has become the most common acquired heart disease in children, and the incidence of KD is gradually increasing, in particular the gradual increase of the incidence of incomplete KD, which complicates the early diagnosis of this disease [5]. Intravenous immunoglobulin (IVIG) combined with aspirin treatment of KD can reduce the incidence of CAL from 15–25% to 5% [6]. Although the use of high-dose IVIG significantly reduces the incidence of CAL, 10% to 20% of children with KD remain nonresponsive to IVIG and have a higher incidence of CAL [7]. The ability to predict KD patients' resistance to IVIG early, to take the appropriate measures for prevention or treatment as soon as possible, is of great significance for reducing CAL and improving prognosis.

KD is associated with immune and genetic susceptibility. In recent years, with the application of genome-wide association analysis, many studies have shown that a correlation between IVIG nonresponsiveness and genetic polymorphisms [8–11]. Genes related to the FcyR and ITPKC pathways have been extensively studied [12, 13]. Studies suggest that Fc receptor-mediated anti-inflammatory effects and calcium-mediated T cell activation do not respond to IVIG. The study by Shreshtha et al. has a certain guiding significance for the prediction and treatment of IVIG nonresponsive KD [14]. Onouchi et al. [15] could successfully and reliably demonstrate the association of the functional SNPs of ITPKC and CASP3 with susceptibility to KD and found that these SNPs cooperatively affect the severity of KD in the Japanese population.

Purinergic receptor P2Y12 (P2RY12) belongs to the family of G-protein-coupled receptors. P2RY12 regulates platelet activation and aggregation and is a potential target for the treatment of thromboembolism and other coagulopathies [16–18]. Many studies have shown that P2RY12 may be a protective factor for clopidogrel and aspirin resistance [19, 20]. As we described previously [21], the P2RY12: rs7637803 TT variant genotype increased the risk of coronary artery aneurysm in KD patients in a southern Chinese population. However, no studies have focused on the connection between the P2RY12 polymorphism and IVIG resistance in KD. Accordingly, in the present study, we selected five single-nucleotide polymorphisms (SNPs) in P2RY12 to investigate the relationship between SNPs and IVIG resistance in patients with Kawasaki disease.

## 2. Materials and Methods

### 2.1. Study Population

In the current study, all KD cases and healthy controls were restricted to unrelated ethnic Chinese Han individuals. A total of 759 patients with KD were recruited mainly from the Department of Pediatric Cardiology of the Guangzhou Women and Children's Medical Center between January 2012 and January 2017. All of the KD cases were classified mainly according to the guidelines for diagnosis, treatment, and long-term management of KD prescribed by the American Heart Association in 2004 [22]. All subjects provided written informed consent signed by their parents. This study was approved by the Institutional Review Board of Guangzhou Women and Children's Medical Center (GZR2015-099).

### 2.2. SNP Selection and Genotyping

We chose the five SNPs (rs9859538 G > A, rs1491974 A > G, rs7637803 C > T, rs6809699 A > C, and rs2046934 G > A) in the *P2RY12* gene. Samples were collected with the instructions of the Genomic DNA Extraction Kit (Tiangen, Beijing, China). The DNA was quantified using a nucleic acid quantifier. The P2RY12 polymorphisms were genotyped with the TaqMan reagent. High-quality DNA samples were genotyped using the Taqman real-time PCR method (Applied Biosystems, Foster City, CA, USA). We performed the Taqman real-time Polymerase chain reaction (PCR) assay to genotype these SNPS [23]. Moreover, 10% of the samples were randomly selected and repeated, and the reproducibility was 100% concordant.

### 2.3. Statistical Analysis

Genotype frequencies of each SNP as well as the demographic variables were compared using the Chi-squared test between the KD cases and the controls. The OR and 95% confidence interval (CI) were calculated by unconditional logistic regression analyses, with adjustments for age and sex. Genotypic frequencies in controls for each SNP were tested for departure from the Hardy–Weinberg equilibrium (HWE) using goodness-of-fit *χ*2 test. We preset 0.2 as the false-positive report probability (FPRP) threshold and chose a prior probability of 0.1 to detect OR of 0.67 (for protective effects). The statistical analyses procedures have been described previously [21].

## 3. Results

### 3.1. Population Characteristics

Table 1 shows the demographic characteristics of KD cases that were resistant or sensitive to IVIG therapy. The IVIG resistance was diagnosed based on diagnosis, treatment, and long-term management of Kawasaki Disease: A Scientific Statement for Health Professionals from the American Heart Association in 2017 [2]. Patients with KD developed recrudescent or persistent fever at least 36 hours after the end of their IVIG infusion, denoted as IVIG resistant. The mean age (±standard deviation (SD)) was 28.2 ± 24.4 months (range 1–116 months) for KD patients who were resistant to IVIG therapy and 26.58 ± 21.86 months (range 1–156 months) for KD patients who were sensitive to IVIG therapy. Briefly, no significant differences were observed in age (28.2 ± 24.4 vs. 26.58 ± 21.86,*P*=0.0672) or gender (*P*=0.5545) between KD patients sensitive or resistant to IVIG therapy. 65.54% of KD patients who were resistant to IVIG therapy were male, and the male ratio was 68.09% in KD patients who were sensitive to IVIG therapy. The proportions of women were 34.46% and 31.91%, respectively.

### 3.2. Associations between P2Y12 Gene Polymorphisms and IVIG Resistance

The genotype distributions of P2RY12 polymorphisms in Kawasaki disease patients with IVIG resistance and IVIG sensitivity are shown in Table 2. There were no significant declinations of the HWE for genotypic frequencies of each SNP (*P*=0.4397 for rs9859538 G > A, *P*=0.3464 for rs1491974 A > G, *P*=0.8563 for rs7637803 C > T, *P*=0.4890 for/rs6809699 A > C, and *P*=0.0690 for rs2046934 G > A).We selected rs9859538 G > A, rs1491974 G > A, rs7637803 C > T, rs6809699 C > A, and rs2046934 A > G of P2RY12 to determine whether there is any relationship between Kawasaki disease patients with IVIG resistance and IVIG sensitivity. Of the five investigated SNPs, a significant difference in the genotype distributions between IVIG resistance and IVIG sensitivity in KD patients was only observed for the rs6809699 A > C polymorphism (*P*=0.0194). After adjusting for age and gender, the carriers of a rs6809699 C allele had an odds ratio (OR) of 0.44 to 0.49 for IVIG sensitivity (AC vs. AA: adjusted OR = 0.48, 95% confidence interval (CI) = 0.27–0.84, *P*=0.011; AC/CC vs. AA: adjusted OR = 0.47, 95% CI = 0.27–0.83, *P*=0.0084) compared to the carriers of a rs6809699 AA genotype, suggesting the protective effect of this SNP against IVIG resistance. However, no association was found for the four remaining polymorphisms. While the protective genotypes of the five SNPs were combined, we found that patients with 4-5 protective genotypes experienced a significantly decreased IVIG resistance compared patients with only 0–3 protective genotypes (adjusted OR = 0.27, 95% CI = 0.13–0.57, *P*=0.0006).

### 3.3. Stratification Analysis

We further explored the association between the P2RY12 gene rs6809699 A > C polymorphism and the combined effects of protective genotypes in KD patients with IVIG resistance in a stratification analysis by age and gender (Table 3). Compared to the rs6809699 CC genotype, the protective effect of the CA/AA genotypes was more predominant in children ≤60 months of age (adjusted OR = 0.42, 95% CI = 0.23–0.76, *P*=0.0045) and males (adjusted OR = 0.39, 95% CI = 0.19–0.81, *P*=0.0115). In addition, combined analysis indicated that the 5 protective genotypes collectively decreased KD patients with IVIG resistance in children ≤60 months of age (adjusted OR = 0.22, 95% CI = 0.10–0.52, *P*=0.0005) and males (adjusted OR = 0.27, 95% CI = 0.11–0.69, *P*=0.0064).

## 4. Discussion

In the current case-control study with 148 KD patients with IVIG resistance and 611 KD patients with IVIG sensitivity, we verified that the P2RY12 gene rs6809699 A > C polymorphism was associated with IVIG resistance in KD patients. To the best of our knowledge, this is the first study to investigate the association between P2RY12 gene polymorphisms and IVIG resistance in KD in Southern Chinese children.

The efficacy of IVIG administered in the acute phase of KD is known to reduce the prevalence of coronary artery abnormalities [24]. Approximately 10% to 20% of patients with KD develop recrudescent or persistent fever at least 36 hours after the end of their IVIG infusion, denoted as being IVIG resistant [25]. Many studies have shown that patients who are resistant to initial IVIG are at an increased risk of developing coronary artery abnormalities [26, 27]. The immunologic basis of IVIG resistance is unknown; however, it is likely that host genetic factors, such as polymorphisms in the Fc gamma receptors, play a role in resistance to IVIG [2]. Weng et al. [8] showed that the IL-1B-511 TT and IL-1B-31 CC genotypes, or the TC/TC diplotype, may be associated with initial IVIG resistance in Taiwanese children with KD. Khor et al. [11] showed that the FCGR 2A locus may have implications for understanding immune activation in KD pathogenesis and the mechanism of response to intravenous immunoglobulin. Shrestha et al. [12, 14] proved that the FcyR IIIB-NA1 gene polymorphism is associated with IVIG resistance. Onouchi et al. [28] found that these SNPs cooperatively affect the IVIG response in the Japanese population.

The efficacy of IVIG administered in the acute phase of KD is known to reduce the prevalence of coronary artery abnormalities [2]. Because aspirin has important anti-inflammatory and antiplatelet activities, it has been used for the treatment of KD for many years. In patients with moderate but not large or giant aneurysms, aspirin therapy may be combined with clopidogrel to antagonize ADP-mediated platelet activation [2]. The purinergic receptor P2RY12 is a clinical target in both cardiovascular and cerebrovascular diseases, since the inhibition of platelet P2RY12 prevents ADP-induced platelet aggregation and thereby reduces the risk of thrombosis [29]. Many studies have shown that the five SNPs (rs9859538 G > A, rs1491974 A > G, rs7637803 C > T, rs6809699 A > C, and rs2046934 G > A) in the *P2RY12* gene are associated with aspirin and clopidogrel resistance in heart disease and increases coronary artery aneurysm risk in Kawasaki disease. Karazhanova et al. [19] found an association between the H2 haplotype in the P2RY12 gene and aspirin resistance in patients with coronary artery disease. Li et al. [20] showed that the rs2046934 C allele may be a protective factor in clopidogrel resistance, rs2046934 was included as one of the variations in the P2RY12 gene that was examined and was found to contribute to interindividual variability during clopidogrel therapy. Lu et al. [21] found that rs7637803 TT variant genotype increases coronary artery aneurysm risk in Kawasaki Disease in a Southern Chinese Population, the rs7637803 genotype might be used as a biomarker to predict the occurrence of coronary artery aneurysm in KD patients. Zhang et al. [30] found that the rs6809699 minor allele could predict bleedings in ST-elevation myocardial infarction patients after percutaneous coronary intervention. Timur et al. [31] showed that rs9859538 was found to be associated with high residual platelet reactivity; the minor allele of rs9859538 was associated with a 2-fold increase in having both ≥20% arachidonic acid-stimulated and ≥70% adenosine diphosphate (10 *μ*M)-stimulated maximal platelet aggregation. In terms of IVIG, aspirin and clopidogrel are the three main drugs for treating KD. However, because the five SNPs in the P2RY12 gene are associated with aspirin and clopidogrel resistance, based on this background, we investigated whether the five SNPS were a relationship between P2RY12 polymorphisms and IVIG resistance in KD patients.

A previous study by our research group focused on the relationship between P2RY12 polymorphism with KD genetic susceptibility and coronary artery aneurysm (CAA) complications [21]. The study found that rs7637803 significantly increased CAA risk in KD patients. Moreover, patients who were resistant to initial IVIG were found to be at an increased risk of developing CAA. As such, we considered whether there was a relationship between P2RY12 polymorphisms and IVIG resistance in KD patients. The present study included a large cohort of KD cases, including 148 KD patients with IVIG resistance and 611 KD patients with IVIG sensitivity from a southern Chinese population. P2RY12 polymorphisms were selected as the study subject, and we verified that the P2RY12 gene rs6809699 A > C polymorphism was associated with IVIG resistance in KD patients. However, no association was found for the four remaining polymorphisms. Future studies should employ a larger sample size in order to further validate our findings.

KD is an age-related disease that generally affects young children aged ≤60 months. Furthermore, the ratio of incidence of the disease in males to females is 1.5 to 1. 2 [2]. However, why Kawasaki disease is more likely to occur in children younger than 5 years old remains unknown. In our study, compared to the rs6809699 CC genotype, the protective effect of the CA/AA genotypes was more predominant in children ≤60 months of age and males, In addition, combined analysis indicated that the 5 protective genotypes collectively decreased KD patients with IVIG resistance in children ≤60 months of age and males, which may be one reason why Kawasaki disease is less common in children aged more than 60 months and why incidence among females is less than that among males. The power of the test is likely diminished for the age ranges of >60 months of age, indicating that rs6809699 CA/AA variant genotypes did not decrease risk of IVIG resistance in children aged more than 60 months.

The present study had several limitations. Although this study is the first investigation in southern Chinese subjects, a number of limitations should be acknowledged. First, although the sample size in the current study was large, it is not enough due to the low incidence rate of IVIG resistance in KD cases. Therefore, multicenter studies with larger sample sizes are needed to confirm the correlation between P2RY12 and IVIG resistance in KD patients. Second, we only adjusted for age and gender in the logistic regression analysis and were not able to collect and control for other factors, such as the influence of family genetic factors and birth history. Future studies with a larger sample size and functional experiments should be conducted to confirm the results obtained in this study. Third, to the best of our knowledge except for the P2RY12 gene polymorphism, there are many other genetic and nongenetic factors that have effect on the risk of IVIG resistance in Kawasaki patients. However, in our current study, we only studied the correlation between P2RY12 gene polymorphism and IVIG resistance in Kawasaki patients and did not involve other genes. Therefore, it is unclear whether the P2RY12 gene affects IVIG resistance in Kawasaki patients together with other genes. In future research, we plan to study the relationships between other genes and IVIG resistance in Kawasaki patients and to further explore whether P2RY12 SNPs are linked to other genes to related to IVIG resistance in Kawasaki patients.

## Figures and Tables

**Table 1:** Characteristics in KD patients resistant or sensitive to IVIG therapy.

Variables	IVIG-R^a^	IVIG-S^b^	*P* value^c^
Age range, month	1–116	1–156	0.0672
Mean ± SD	28.20 ± 24.40	26.58 ± 21.86	
≤60	134 (90.54%)	579 (94.76%)	
>60	14 (9.46%)	32 (5.24%)	
Gender			0.5545
Male	97 (65.54%)	416 (68.09%)	
Female	51 (34.46%)	195 (31.91%)	

^a^KD patients resistant to IVIG therapy. ^b^KD patients sensitive to IVIG therapy. ^c^Two-sided *χ*^2^ test for the distribution between KD patients with resistance or sensitivity to IVIG.

**Table 2:** Genotype frequency distribution of P2RY12 polymorphisms in KD patients resistant or sensitive to IVIG therapy.

Genotype		IVIG R (*n* = 148)	IVIG S (*n* = 611)	*P* value^a^	OR (95% CI)	*P* value	Adjusted OR (95% CI)	*P* value^b^
*P2RY12/rs9859538 G > A HWE = 0.4397*								
GG		106 (71.62%)	444 (72.67%)	0.8268	1.00		1.00	
GA		37 (25.00%)	152 (24.88%)		1.02 (0.67–1.55)	0.9273	1.02 (0.67–1.55)	0.9327
AA		5 (3.38%)	15 (2.45%)		1.40 (0.50–3.93)	0.5267	1.41 (0.50–3.98)	0.5130
Dominant		42 (28.38%)	167 (27.33%)	0.7982	1.05 (0.81–1.57)	0.7983	1.05 (0.71–1.57)	0.7992
Recessive		143 (96.62%)	596 (97.55%)	0.5419	1.39 (0.50–3.89)	0.5306	1.41 (0.50–3.94)	0.5164
*P2RY12/rs1491974 A > G HWE = 0.3464*								
AA		46 (31.08%)	180 (29.46%)	0.9124	1.00		1.00	
AG		75 (50.68%)	313 (51.23%)		0.94 (0.62–141)	0.7584	0.95 (0.63–1.43)	0.8003
GG		27 (18.24%)	118 (19.31%)		0.90 (0.53–1.52)	0.6821	0.90 (0.53–1.52)	0.6866
Dominant		102 (68.92%)	431 (70.54%)	0.6997	0.93 (0.63–1.37)	0.6988	0.93 (0.63–1.38)	0.7313
Recessive		121 (81.76%)	493 （80.69%)	0.7655	0.93 (0.59–1.48)	0.7666	0.93 (0.58–1.47)	0.7486
*P2RY12/rs7637803 C > T HWE = 0.8563*								
CC		109 (73.65%)	433 (70.87%)	0.7686	1.00		1.00	
CT		36 (24.32%)	162 (26.51%)		0.88 (0.58–1.34)	0.5586	0.88 (0.58–1.34)	0.5593
TT		3 (2.03%)	16 (2.62%)		0.75 (0.21–2.60)	0.6445	0.75 (0.21–2.62)	0.6515
Dominant		39 (26.35%)	178 (29.13%)	0.4990	0.87 (0.58–1.31)	0.5019	0.87 (0.58–1.31)	0.5040
Recessive		145 (97.97%)	595 (97.38%)	0.6715	0.77 (0.22–2.68)	0.6803	0.77 (0.22–2.69)	0.6871
*P2RY12/rs6809699 A > C HWE = 0.4890*								
AA		132 (89.19%)	487 (79.71%)	0.0194	1.00		1.00	
AC		15 (10.14%)	116 (18.99%)		0.48 (0.27–0.85)	0.011	0.48 (0.27–0.84)	0.0110
CC		1 (0.68%)	8 (1.31%)		0.46 (0.6–3.72)	0.468	0.44 (0.06–3.58)	0.4449
Dominant		16 (10.81%)	124 (20.29%)	0.0050	0.48 (0.27–0.83)	0.009	0.47 (0.27–0.83)	0.0084
Recessive		147 (99.32%)	603 (98.69%)	0.4949	0.51 (0.06–4.13)	0.5304	0.49 (0.06–3.99)	0.5069
*P2RY12/rs2046934 G > A HWE = 0.0690*								
GG		114 (77.03%)	443 (72.50%)	0.4278	1.00		1.00	
GA		29 (19.59%)	150 (24.55%)		0.75 (0.48–1.18)	0.2107	0.75 (0.48–1.67)	0.2002
AA		5 (3.38%)	18 (2.95%)		1.08 (0.39–2.97)	0.8823	1.07 (0.39–2.95)	0.8946
Dominant		34 (22.97%)	168 (27.50%)	0.2583	0.79 (0.52–1.20)	0.2648	0.78 (0.51–1.19)	0.2513
Recessive		143 (96.62%)	593 (97.05%)	0.7859	1.15 (0.42–3.12)	0.7832	1.15 (0.42–3.14)	0.7927
Combined effect of protective genotypes							
0–3		140 (94.59%)	507 (82.98%)	<0.0001	1.00		1.00	
4–5		8 (5.41%)	104 (17.02%)		0.27 (0.13–0.57)	0.0006	0.27 (0.13–0.57)	0.0006

^a^Two-sided *χ*^2^ test for distributions between Kawasaki disease patients with IVIG resistance and IVIG sensitivity. ^b^Adjusted for age and gender status in logistic regress models.

**Table 3:** Stratification analysis of P2RY12 polymorphisms in KD cases who were resistant or sensitive to IVIG therapy.

Variable	rs6809699 (IVIG R/IVIG S)		*P* value^a^	OR (95% CI)	*P* value	Adjusted OR (95% CI)	*P* value^b^	Combined analysis^c^ (IVIG R/IVIG S)		*P* value^a^	OR (95% CI)	*P* value	Adjusted OR (95% CI)	*P* value^b^
	AA	AC/CC						0–3	4–5					
Age, months														
≤60	121/460	13/119	**0.0019**	0.42 (0.23–0.76)	**0.0045**	0.42 (0.23–0.76)	**0.0045**	128/479	6/100	**<0.0001**	0.23 (0.10–0.52)	**0.0005**	0.22 (0.10–0.52)	**0.0005**
>60	11/27	3/5	0.6378	1.47 (0.30–7.25)	0.6340	1.39 (0.28–6.99)	0.6898	12/28	2/4	0.8695	1.17 (0.19–7.25)	0.8687	1.02 (0.16–6.55)	0.9805
Gender														
Male	88/330	9/86	**0.0055**	0.39 (0.19–0.81)	**0.0115**	0.38 (0.19–0.80)	**0.0100**	92/347	5/69	**0.0014**	0.27 (0.11–0.70)	**0.0066**	0.27 (0.11–0.69)	**0.0064**
Female	44/157	7/38	0.3302	0.66 (0.28–1.57)	0.3464	0.66 (0.27–1.57)	0.3458	48/160	3/35	**0.0202**	0.29 (0.08–0.97)	**0.0466**	0.28 (0.08–0.96)	**0.0430**

^a^Two-sided *χ*^2^ test for distributions between Kawasaki disease patients with IVIG resistance and IVIG sensitivity. ^b^Adjusted for age and gender, stratified by gender (adjusted for age) and age (adjusted for gender). ^c^Combined effect of protective genotypes.

## Data Availability

The data used to support the findings of this study are included within the article.
